# GOATOOLS: A Python library for Gene Ontology analyses

**DOI:** 10.1038/s41598-018-28948-z

**Published:** 2018-07-18

**Authors:** D. V. Klopfenstein, Liangsheng Zhang, Brent S. Pedersen, Fidel Ramírez, Alex Warwick Vesztrocy, Aurélien Naldi, Christopher J. Mungall, Jeffrey M. Yunes, Olga Botvinnik, Mark Weigel, Will Dampier, Christophe Dessimoz, Patrick Flick, Haibao Tang

**Affiliations:** 1 School of Biomedical Engineering, Science, and Health Systems, Drexel University, Philadelphia, PA USA; 2 Center for Genomics and Biotechnology, Fujian Agriculture and Forestry University, Fuzhou, China; 3 Department of Human Genetics, University of Utah, Salt Lake City, UT USA; 4 Max Planck Institute of Immunobiology and Epigenetics, Freiburg, Germany; 5 Department of Genetics, Evolution and Environment, University College London, London, UK; 6 Center for Integrative Genomics, Faculty of Biology and Medicine, University of Lausanne, Lausanne, Switzerland; 7 Division of Environmental Genomics and Systems Biology, Lawrence Berkeley National Laboratory, Berkeley, CA USA; 8 UC Berkeley - UCSF Graduate Program in Bioengineering, University of California, San Francisco, CA USA; 9 Bioinformatics and Systems Biology Program, University of California, San Diego, CA USA; 10 Independent Researcher, Philadelphia, PA USA; 11 Department of Microbiology and Immunology, Drexel University College of Medicine, Philadelphia, PA USA; 12 School of Computational Science and Engineering, Georgia Institute of Technology, Atlanta, GA USA

## Abstract

The biological interpretation of gene lists with interesting shared properties, such as up- or down-regulation in a particular experiment, is typically accomplished using gene ontology enrichment analysis tools. Given a list of genes, a gene ontology (GO) enrichment analysis may return hundreds of statistically significant GO results in a “flat” list, which can be challenging to summarize. It can also be difficult to keep pace with rapidly expanding biological knowledge, which often results in daily changes to any of the over 47,000 gene ontologies that describe biological knowledge. GOATOOLS, a Python-based library, makes it more efficient to stay current with the latest ontologies and annotations, perform gene ontology enrichment analyses to determine over- and under-represented terms, and organize results for greater clarity and easier interpretation using a novel GOATOOLS GO grouping method. We performed functional analyses on both stochastic simulation data and real data from a published RNA-seq study to compare the enrichment results from GOATOOLS to two other popular tools: DAVID and GOstats. GOATOOLS is freely available through GitHub: https://github.com/tanghaibao/goatools.

## Introduction

Gene ontology enrichment analysis (GOEA) is used to test the overrepresentation of gene ontology terms in a list of genes or gene products in order to understand their biological significance. Members of the Gene Ontology Consortium (GOC)^1^ from all over the world collaborate to develop the Gene Ontology (GO), a resource to describe the molecular function, cellular localization, and biological processes of gene products across multiple species. The ontology includes over 47,000 terms (as of April 2018) and describes formal relationships among them. GOC members annotate ontology terms to specific gene products on the basis of experimental and computational prediction^2^. Annotation coverage of GO terms to individual genes is high for humans and model organisms, with 85% of 20 k human protein-coding genes having GO annotations, 90% of the 22 k Ensembl mouse genes, and 77% of the 14 k fly genes. Both ontologies and annotations can change incrementally on a daily basis^3^. To keep a laboratory’s many functional genomic studies up-to-date with the rapidly evolving biological knowledge, it can be helpful to use a programmatic API built directly into an analysis pipeline; GOATOOLS does just that.

Python has a large, diverse open-source development community and comprehensive scientific computing libraries for building robust and reproducible computational workflows. GOATOOLS allows GO term manipulation, GOEA testing, and custom ontology visualization in gene functional studies.

We describe the GOATOOLS implementation first, followed by stochastic simulations, and finally demonstrate a case study using gene expression data from the paper by Gjoneska *et al*. (2015), *Conserved epigenomic signals in mice and humans reveal immune basis of Alzheimer*’*s disease*^4^. Going forward, we will refer to this paper as the GP paper for the two first authors, Gjoneska and Pfenning. We then compare GOATOOLS results with two mainstream methods: the web-based DAVID tool (Database for Annotation, Visualization, and Integrated Discovery)^5^ and the R library, GOstats^6^. We demonstrate that GOATOOLS yields similar or better GOEA results and provides more flexibility via the Python API to group, sort, summarize, and visualize the results.

## Materials and Methods

### GOATOOLS development

GOATOOLS is open-source and available on GitHub (https://github.com/tanghaibao/goatools). In the tradition of open-source packages, GOATOOLS releases are early and often and have undergone continuous refinement over the last seven years. GOATOOLS was successfully used to explore a variety of research questions concerning a wide range of organisms, including over twenty different plant species, about ten fish species, five animal species, fungus, bacteria, and microalgae^7^. One publication that used and cited GOATOOLS investigated the immunogenetics of disease resistance of the common carp (*Cyprinus carpio*), an important aquacultured fish^8^. Another publication used GOATOOLS to study the maternal-to-zygotic transition of an embryo^9^.

### GOATOOLS implementation

GOATOOLS can be installed through package managers including Python *easy_install* or *pip*, and is also available as a bioconda package (https://bioconda.github.io). Extensive tutorials and Jupyter notebooks are available to demonstrate the usage of GOATOOLS in step-by-step fashion. In the following section, we detail the GOATOOLS implementation, including details on file I/O, data structure, statistical testing, reporting, and visualization.

### File I/O and data structure

A GOEA requires both a copy of the ontology, which describes terms and relationships among them, and a set of annotations, which associates the GO terms to specific gene products. The ontology is available from the main GO website (http://geneontology.org/page/download-ontology). There are three versions of the GO ontology: GO-basic, GO, and GO-plus, among which only GO-basic is guaranteed to be acyclic^10^. GOATOOLS traverses the ontology, which is stored as a graph, and thus requires the acyclic version found in GO-basic; this is the GO version recommended for most GO-based annotation tools^10^.

Most ontology systems are also becoming available in a JSON (JavaScript Object Notation) format, which is a lightweight, language independent interchange format^11^. The JSON file currently available is *go-plus*.*json*, which contains more extensive information than the smaller *go-basic*.*obo*. Accounting for the larger size of the more information-rich GO-plus file, the rate of reading and parsing the ontologies from the JSON file is about three times faster than the rate of reading the obo text file.

The annotations are currently available for download from the GOC as GO Annotation Format (GAF), from NCBI’s FTP server in a gene2go format, or from the European Bioinformatics Institute’s FTP site in the Gene Product Association Data format (GPAD). GOATOOLS can efficiently parse these relevant file formats to retrieve rich attributes of each term and model the term relationship using the *is_a* attribute as well as *part_of*, and *regulates* relationships into a directed acyclic graph (DAG)^12^. The DAG data structure allows traversal of terms along the hierarchy for tasks such as determination of level or depth, retrieval of parent or child terms, and calculation of semantic similarities (e.g. Resnik’s score^13^ and Lin’s score^14^) between terms. Mapping between regular GO terms and a restricted subset of GO (GO slims) is also supported.

GOATOOLS returns GOEA results in a variety of formats: EXCEL spreadsheet, tab-separated text file, JSON file, or Python variable containing a list of results with the GO results grouped by function as part of the API.

### Statistical Testing

Many functional genomics studies look to see if any selected gene sets contain enrichment (or perhaps less common, under-representation) of certain functional classes, which is a critical goal in the study of differential gene regulation^1^. The frequency of genes for a particular GO term in the sample is compared to the frequency in the background. A P-value is then computed, often on the basis of Fisher’s exact test^15^. Of the 68 GOEA tools reviewed by Huang *et al*., 20 support Fisher’s exact test, which uses a hypergeometric distribution during the calculation. The raw hypergeometric test is also popular with 21 tools for determining uncorrected P-values. Tests seen in other tools include chi-square test, t-test, Z-score, and Kolmogorov-Smirnov test^16^. GOATOOLS currently uses the Fisher’s exact test to compute uncorrected P-values. Many users preferred Fisher’s exact test over, for example, the chi-square test because Fisher’s exact test is more accurate^17^. Another popular tool, DAVID, uses Fisher’s exact test, along with a modified EASE score^5^. The review by Rivals *et al*. discusses trade-offs for various statistical tests specifically for testing the enrichment of GO terms^18^.

Due to a large number of tests performed, the individual P-value should be corrected to control the false positive rates^19^. GOATOOLS contains a large collection of multiple test correction procedures (12 tests to date), which include all the functions available from the statsmodels Python library^20^. Each of these tests may be more appropriate when used under specific experimental settings or if able to offer different levels of stringency. We have implemented popular methods including Bonferroni, Sidak, and Holm, as well as False Discovery Rate (FDR) procedures such as Benjamini-Hochberg or resampling-based FDR^19^.

As an example, to demonstrate why a researcher may want to choose one kind of multiple test correction over another, we consider two popular tests: Bonferroni, which controls the family wise error rate (FWER), and Benjamini/Hochberg, which controls the false discovery rate (FDR). The FWER is the probability that there will be at most one false positive. Thus, a FWER set at 0.05 means that there is a 5% chance that there will be even one false positive. The FDR quantifies the fraction of discoveries that are allowable as false positives. A FDR set to 0.05 means that we have accepted that up to 5% of our “statistically significant” results may be false positives.

The Bonferroni results are guaranteed to have fewer false positives than the FDR tests. But the drawback is that Bonferroni is extremely conservative, with the loss of statistical power resulting in many missed true positives. In other words, truly significant observations are discarded. FDR provides more true positive results overall, with the downside of more false positives up to a maximum percentage of discoveries set by the researcher. FWER corrections like Bonferroni are desirable if a conclusion drawn from all ontology P-values for a set of genes would be invalidated if at least one of the P-values shows significance when there is none. Such strictness may not be desirable. For example, the conclusion, “a set of genes is rich in immune functions,” is valid when many gene ontology tests correctly show significance for immune functions, but one test incorrectly shows significance for one specific immune function.

FDR controls have been recommended over Bonferroni-type multiple test corrections in health studies^21^. A recent paper by Goeman and Solari focuses on the trade-offs of the various multiple hypothesis tests^22^. The exhaustive list of statistical tests supported by GOATOOLS can be found at the GOATOOLS website (https://github.com/tanghaibao/goatools#available-statistical-tests-for-calculating-uncorrected-P-values).

### Reporting

Gene Ontology Enrichment Analysis tools, when given a list of genes, can return hundreds of statistically significant GO results in a “flat” list, which can be challenging to summarize or to discern from a systems perspective using only a basic sort, like sorting all results by P-value. A “flat” list is a list of GO terms not organized with any consideration to the innate hierarchy that the GO terms have with one another^23^.

The researcher may wish to retain all of the GOEA results, but re-organize them under general sections, like *immune* or *viral/bacteria*. In a flat list of GO terms sorted by P-value, GO terms related to interesting groups, like *immune* or *viral/bacteria*, may be scattered throughout the list (Table 1A). Additionally, in a “flat” list it can be difficult to identify other general groups besides *immune* and *viral/bacteria* that might be present when the GO terms of various potential groups are interleaved among one another.

**Table 1:** The output of GOATOOLS GO grouping is a list.

A) Ungrouped GO IDs sorted by P-value		
GO Name	P-value	
immune system process	3.74E-07	
defense response to protozoan	5.56E-06	
defense response to virus	2.93E-04	
positive regulation of extrinsic apoptotic signaling pathway	5.94E-04	
positive regulation of T cell mediated cytotoxicity	7.30E-04	
response to bacterium	7.30E-04	
+reg. of cysteine-type endopeptidase activity in apoptotic process	1.35E-02	
pyroptosis	1.86E-02	
positive regulation of I-kappaB kinase/NF-kappaB signaling	3.15E-02	
+reg. of tumor necrosis factor-mediated signaling pathway	3.70E-02	
antigen processing and presentation of exogenous antigen	4.32E-02	
purinergic nucleotide receptor signaling pathway	4.32E-02	
**B) Grouped GO IDs sorted by P-value**		
**GO Name**	**P-value**	**Section**
positive regulation of extrinsic apoptotic signaling pathway	5.94E-04	cell death
+reg. of cysteine-type endopeptidase activity in apoptotic process	1.35E-02	cell death
pyroptosis	1.86E-02	cell death
mmune system process	3.74E-07	immune
positive regulation of T cell mediated cytotoxicity	7.30E-04	immune
antigen processing and presentation of exogenous antigen	4.32E-02	immune
positive regulation of I-kappaB kinase/NF-kappaB signaling	3.15E-02	signaling
+reg. of tumor necrosis factor-mediated signaling pathway	3.70E-02	signaling
purinergic nucleotide receptor signaling pathway	4.32E-02	signaling
defense response to protozoan	5.56E-06	viral/bacteria
defense response to virus	2.93E-04	viral/bacteria
response to bacterium	7.30E-04	viral/bacteria

GOATOOLS grouping makes GO lists easier to read. Even a short list of GO terms can be hard to to read and difficult to discern which GO terms might be related (A). GOATOOLS grouping makes results easier to read (B). GO terms in Table A are sorted by P-value. In Table B, GO terms are grouped first and then sorted by P-value. These tables were produced using GOATOOLS grouping and table writing code. The first column, ‘GO Name’ is the name of the GO term found in the GO DAG. The second column shows the P-value obtained from running a GOEA analysis. The third column shows the functional group containing the GO term, called a ‘section name’. Normally GO grouping lists are black and white, but a table can be colorized (shown) if provided with a Python dictionary where the key is the section and the value is the color. The creation of section names for grouping is described in detail in the text.

GOATOOLS grouping allows users to display GO terms and their associated study genes returned from GOEAs under general sections. GO terms in each section may then be sorted by P-value to easily see both the most statistically significant terms in *immune* and the most statistically significant GO terms in *viral/bacteria* (Table 1B). The user can then reduce this list to produce a short summary list by printing only the top N sorted GO terms in each section, where N is a small number such as 1, 2, or 3.

### Gene ontology graph layout

As of April 2018, the DAG contains over 47,000 GO terms and is divided into three major branches with each branch emanating from a single GO parent term at the top-level fanning out to over 28,000 of GO terms at the bottom level. The three broad top-level branch terms are *biological_process*, *molecular_function*, and *cellular_component*. GO terms may have more than one parent. There are over 20 GO children directly under the top-level branch term, *biological_process* (Table 2).

**Table 2:** The descendant counts of GO terms at depth-01 are highly skewed.

D1 Alias	dcnt	depth	GO	name
	29,625	D00	GO:0008150	biological_process
A	18,703	D01	GO:0009987	cellular process
B	13,064	D01	GO:0065007	biological regulation
C	9,805	D01	GO:0008152	metabolic process
D	7,544	D01	GO:0032501	multicellular organismal process
E	6,473	D01	GO:0032502	developmental process
F	6,004	D01	GO:0050896	response to stimulus
G	4,354	D01	GO:0051179	localization
H	3,572	D01	GO:0071840	cellular component organization or biogenesis
I	2,369	D01	GO:0051704	multi-organism process
J	2,310	D01	GO:0023052	signaling
K	1,796	D01	GO:0002376	immune system process
L	1,277	D01	GO:0000003	reproduction
M	1,219	D01	GO:0022414	reproductive process
N	843	D01	GO:0040011	locomotion
O	492	D01	GO:0008283	cell proliferation
P	432	D01	GO:0040007	growth
Q	350	D01	GO:0022610	biological adhesion
R	280	D01	GO:0007610	behavior
S	113	D01	GO:0001906	cell killing
T	72	D01	GO:0044848	biological phase

The root term, *biological_process* has over twenty GO children at depth-01 shown in the table sorted by their number of descendants (dcnt) with *cellular process* at the top having over 18,000 descendants and *cell killing* near the bottom having just over 100 descendants. The first column (D1 Alias) contains a letter used as an alias for each depth-01 GO term. The second column represents the total number of descendants from the specified GO term down to all of its leaf-level GO terms, which have no child GO terms. The third column, depth, shows the root term is at depth-00 and its children are at depth-01. The forth column, GO, is the ID for the term. The fifth column shows the human-readable name of the GO term. GO DAG relationships like *part_of* are used to count descendant counts in this table.

Letters like A, B, and C in the ‘D1 Alias’ column of Table 2 are aliases for depth-01 GO terms. The depth-01 aliases are used to provide the general location in the GO DAG of any one GO term. For example, in Table 2, ‘Q’ is the alias for *biological adhesion*. Immune GO terms descended from *biological adhesion* will have a ‘Q’ associated with them and include *lymphocyte aggregation* (Q), *positive regulation of gamma-delta T cell differentiation* (ABDEKQ), and *positive regulation of activated T cell proliferation* (ABKOQ). The aliases in the letters match the letters in Table 2 and are automatically created by the GOATOOLS code and included in the default printing format.

**Figure 1:**
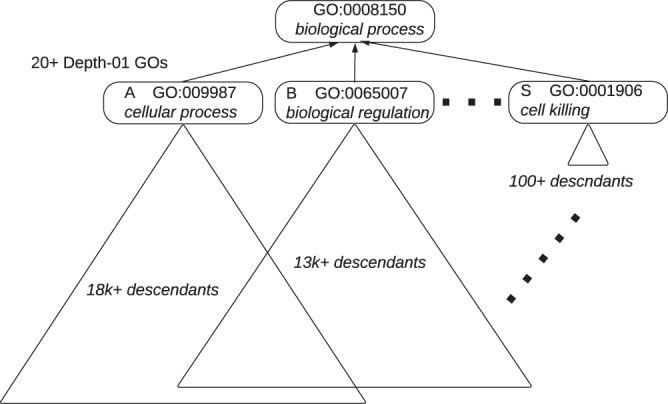
The GO terms at at depth-01 have highly skewed numbers of descendants from *cellular process* which has over 18,000 descendants down to *cell killing* having just over 100 descendants shown here. GO terms within the overlapping triangles descend from both *cellular process* and *biological regulation*. The letters A, B, and S are aliases for the depth-01 GO terms as shown in Table 2. The ellipsis indicate that there are GO terms between *biological regulation* and *cell killing* that are omitted in the figure, but are shown in Table 2.

The number of descendants (descendant counts or *dcnt*) of each of the depth-01 GO children are dramatically skewed and have many shared parents. For example, as of 2018, the top term, biological_process, has over 29,000 descendant GO terms beneath it. The depth-01 GO term, *cellular_process*, just under the top term has more than 18,000 descendants while depth-01 *cell killing* has more than 100 descendants (Fig. 1 and Table 2). This illustrates that the descendant counts are highly skewed among all depth-01 terms. This sort of imbalance is seen throughout the DAG, not just at depth-01 (Table 3).

**Table 3:** The counts of GO terms at all levels and depths is highly skewed across all three branches of the GO.

GO Counts go-basic.obo Apr 4, 2018 47,216 Terms						
Depth or Level	Depth			Level		
	BP	MF	CC	BP	MF	CC
00	1	1	1	1	1	1
01	29	15	21	29	15	21
02	265	126	341	422	152	738
03	1270	527	499	2206	842	1077
04	2374	1515	732	4853	2068	1353
05	3697	4754	901	7310	4971	684
06	4468	1880	790	7215	1993	228
07	4687	1001	584	4658	776	63
08	4221	593	251	2003	207	10
09	3507	328	51	647	84	1
10	2401	160	4	244	13	0
11	1509	135	1	38	19	0
12	826	42	0	0	0	0
13	291	35	0	0	0	0
14	62	21	0	0	0	0
15	14	7	0	0	0	0
16	4	1	0	0	0	0

The deepest GO in the BP branch is at depth 16, while the deepest GO in the CC branch is depth 11. The GO roots are BP (*biological_process*), MF (*molecular_function*), and CC (*cellular_component*). The maximum length path from the root node down to the GO node is *Depth*. The minimum length path is *Level*.

**Figure 2:**
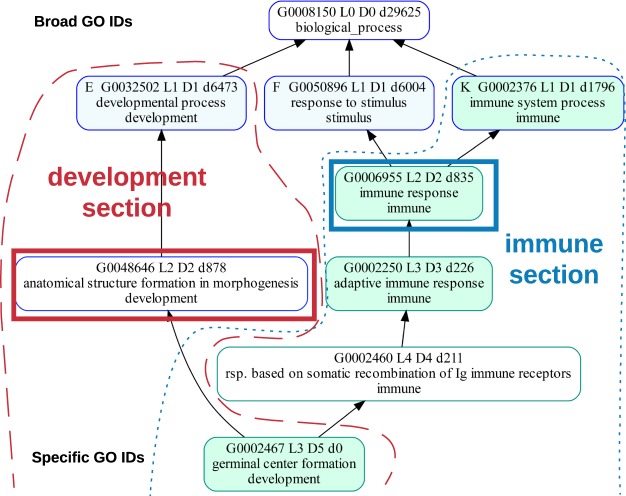
The GO term *germinal center formation* (green term on the bottom) can be in the *development* section (surrounded by dashed red line), the *immune* section (surrounded by the blue dotted line). Section membership for each GO ID is also specified by the text at the bottom of each GO term box. By default, there are five potential GO headers for *germinal center formation* (GO terms with a blue border). The *germinal center formation* is initially in the *development* section because the GO header *anatomical structure formation in morphogenesis* (boxed in red) has a dcnt of 878 while the next lowest GO header is *immune system response* with a dcnt of 1796. But if *immune response* (boxed in blue) is added as a new GO header to the *immune* section, it pulls *germinal center formation* into the *immune* section. The text at the top of each GO term box is described as follows. The total number of GO terms below the GO term box is indicated by the number next to the ‘d’. Level, the minimum path from the top root term is indicated by the number next to the L. Depth, the maximum path from the top root term is indicated by the number next to the D. For example “L3 D5” on *germinal center formation* indicates that the minimum path is 3 (through the through the development section) and the maximum path is 5 (through the immune section).

Because of the highly skewed nature of the ontology graph, level or depth values cannot be used to estimate how close a GO term is to the bottom of the DAG^24^. Given a set of annotations, specificity of a GO term can be estimated from its association information content *tinfo* = −log (frequency), where frequency is the number of associations for the current GO term divided by the total number of associations in the full branch^25^. If no annotations are provided, using descendant counts (*dcnt*) under a GO term worked well in practice as an estimate for defining how specific the GO term is, meaning how close it is to the bottom of the DAG. For example, it can be estimated that terms with thousands of descendants in the DAG, like *developmental process* with an information content (*tinfo*) of 5 and its over 6,000 descendants, are considered broader. Terms at the bottom of the DAG, like *germinal center formation* with a *tinfo* of almost 12 and having no descendants, are considered more specific (Fig. 2). GO terms with a descendant count of zero are at the bottom, or leaf-level of the DAG.

### Grouping method

Our novel approach to GO grouping retains all of the original GO IDs resulting from GOEAs, but rearranges the list so that the results are easier to read or print. The GOATOOLS method for grouping GO IDs uses two steps. The first grouping step uses broad GO terms as GO headers, where the contents of the group under a GO header are the header’s descendant GO terms that are also GOEA results. The second grouping step uses researcher-created section titles like *immune* and *neurological* as section headers, where the contents of each section are GO headers from the first step.

#### Two-step grouping method

The default list of GO headers used in the first step of the GOATOOLS grouping is the list of species-agnostic generic GO slims from the Gene Ontology Consortium (http://www.geneontology.org/ontology/subsets/goslim_generic.obo). As of April 2018, there are over 200 GO IDs in the GOC’s generic GO slim list out of the over 47,000 GO IDs in the full *go-basic*.*obo*. To take full advantage of GOATOOLS grouping, researchers will likely want to add additional broader GO terms as GO headers.

Having user-created section titles used in the second step is necessary because not all terms that researchers may want to view together lie in one GO branch. For example, *immune system process* and *T cell apoptotic process* are in parallel branches that only intersect at the topmost term, *biological_process*. Without a sections list, the GO terms associated with both of these GO headers could end up in separate areas of the results list. The sections list ensures that all GO IDs under both GO headers of interest appear in one area of the grouped GOEA results list. Researchers may also use the sections list to group GO IDs from under different top-level branches, like *biological_process* and *molecular_function*.

GO terms frequently have multiple parents. To print a GO term just once, regardless of how many parents it has, GOATOOLS chooses the “most specific” GO header parent under which to place the GO if it has multiple parents. The user may override this default by adding new GO headers to the sections list. The “most specific” GO header can be determined using either information content (tinfo) or descendants count (dcnt). A user-created function may also be used to determine the most specific header GO term.

#### Assigning sections

Section names are user-specified descriptive text, not GO terms, created using research questions or based on interesting GO terms found enriched in the GOEAs.

For example, the research questions in the GP paper involved immune and neurological functions. So we create two sections, *immune* and *neuro*. Because the GOEA results for the GP data showed a number of GO terms related to virus and bacteria, we added a *virus/bacteria* section.

We created one sections file by grouping the more than 800 total GO terms found significant from the combined findings of GOATOOLS, DAVID6.7, DAVID6.8, and GOstats GOEA runs using all GP study gene sets. After this single sections file is generated, it is then reused twenty times: once for each of the five GP clusters showing significant GO terms for all four tools.

#### Assigning sections details

To begin creating the sections file for the entire project, we run the *wr_sections* script on the list of the 800+ project GO terms, stored in *goids_all*.*txt*. An initial sections file *sections_in*.*txt* is written because there was none to be read.

**$ wr_sections.py goids_all.txt**

hdr GOs(0 in 0 sections, 61 unused) WROTE: sections_in.txt

hdr GOs(0 in 0 sections, 61 unused) WROTE: sections.txt

usr GOs(0 in 0 sections, 840 ungrpd) WROTE: grouped_gos.txt

840 user GO IDs

Three files are written for this initial run:

1. ***sections_in***.***txt*** This file is read if it exists and written if it does not exist. If it is written, all GO headers which currently represent the GO IDs listed in *goids_all*.*txt* are written into the ungrouped area listed under *Misc*. In our studies, 61 GO headers represent the 800+ GO terms.

2. ***sections***.***txt*** This file is always written. It is *sections_in*.*txt*, but with the ungrouped section (*Misc*.) recalculated and all GO IDs annotated with depth, dcnt, name, etc.

3. ***grouped gos***.***txt*** This file is always written. It contains the current grouping of the user GO IDs as guided by *sections_in*.*txt*. On the first run, zero user GO terms are grouped and 840 GO terms are ungrouped.

The initial *sections in*.*txt* contains a GO ID related to immune, *immune system process*, in the ungrouped area:

# SECTION: Misc.

# GO ID NS hdrusr # user dcnt level depth GO name

---------- --- ----- ------ ----- ----- ----- --------------------

GO:0008150 # BP ** 1 uGOs 29625 L00 D00 biological process

GO:0009987 # BP ** 7 uGOs 18703 L01 D01 cellular process

…

GO:0002376 # BP ** 79 uGOs 1796 L01 D01 immune system process

…

To begin to group the immune GO IDs, add a new section *immune* in the *sections_ini*.*txt* and move the *immune system process* GO term into the new section:

**# SECTION: immune**

GO:0002376 # BP ** 79 uGOs 1796 L01 D01 immune system process

# SECTION: Misc.

# GO ID NS hdrusr # user dcnt level depth GO name

---------- --- ----- ------ ----- ----- ----- --------------------

GO:0008150 # BP ** 1 uGOs 29625 L00 D00 biological process

GO:0009987 # BP ** 7 uGOs 18703 L01 D01 cellular process

…

Moving this one GO header, *immune system process* into the *immune* section in *sections_in*.*txt* causes the *wr_sections* script to move 79 of the 800+ into the immune section:

$ wr_sections.py goids_all.txt

hdr GOs(1 in 1 sections, N/A unused) READ: sections_in.txt

hdr GOs(1 in 1 sections, 60 unused) WROTE: sections.txt

usr GOs(79 in 1 sections, 761 ungrpd) WROTE: grouped_gos.txt

GO header and sections decisions can also be made based upon the current GO grouping in *grouped_gos*.*txt*. For example, the *grouped_gos*.*txt* file shows many GO IDs related to *interleukin* and *interferon* are ungrouped. These GO terms fall under the broad GO:0001816, *cytokine production*. Adding GO:0001816 under the *immune* section adds it as a new GO header.

# SECTION: immune

GO:0002376 # BP ** 79 uGOs 1796 L01 D01 immune system process

**GO:0001816 # cytokine production**

# SECTION: Misc.

# GO ID NS hdrusr # user dcnt level depth GO name

---------- --- ----- ------ ----- ----- ----- --------------------

GO:0008150 # BP ** 1 uGOs 29625 L00 D00 biological process

GO:0009987 # BP ** 7 uGOs 18703 L01 D01 cellular process

…

Adding *cytokine production* results in the placement of 19 additional user GO IDs into the *immune* section for a total of 98 grouped GO IDs:

$ wr_sections.py goids_all.txt

hdr GOs(2 in 1 sections, N/A unused) READ: sections_in.txt

hdr GOs(2 in 1 sections, 60 unused) WROTE: sections.txt

usr GOs(98 in 1 sections, 742 ungrpd) WROTE: grouped_gos.txt

To discover that *cytokine production* may be the appropriate GO header to represent *interferon* and *interleukin*, the GO DAG can be queried either by creating a plot or a report. To create a plot containing user relevant GO IDs:

# 1) CREATE A PLOT CONTAINING interferon & interleukin GO IDs

# Create a list GO IDs that match ‘interleukin’ or ‘interferon’

**$ grep inter grouped_gos.txt > gos_inter.txt**

# Plot the list of GO IDs

A

**$ go_plot.py -i gos_inter.txt -o inter.png --sections=sections.txt**

To create a report of the GO Terms up the hierarchy starting from GO:0032611, *interleukin-1 beta production*, up to the root term GO:008159, *biological process*:

# 2) REPORT GO:0032611 "interleukin-1 beta production" to root

**$ wr_hier.py --up GO:0032611**

- GO:0008150 29625 D00 biological_process

-- GO:0032501 7544 D01 multicellular organismal process

--- GO:0001816 110 D02 cytokine production

---- GO:0032612 2 D03 interleukin-1 production

> ----- GO:0032611 0 D04 interleukin-1 beta production

From the printed report, *cytokine production* in the middle of the hierarchy list is a good term to represent bottom term, *interleukin-1 beta production*, because *interleukin-1 production* just below *cytokine production* is too specific and *multicellular organismal process* just above is too broad.

In our example, moving just two GO headers, *immune system process* and *cytokine production*, resulted in the placement of 98 user GO IDs into the *immune* section. Most GO header movements will result in smaller numbers of user GO IDs grouped, but the method remains the same.

#### Researcher-guided grouping method

The human element of a researcher’s subjective input by grouping and describing GO terms can lead to visualizing information in a unique way, which can lead to unexpected insights.

One reason for the need for the researcher’s insight is that a GO term can be accurately described using multiple, and potentially subjective interpretations. For example, *germinal center formation* may be correctly described as being both a *developmental process* and also related to the *adaptive immune response* (Fig. 2).

Germinal centers are a *developmental process* because they are transient structures that develop in the sites of secondary lymphatic organs, such as lymph nodes, during an immune response^26^. Germinal centers are an *adaptive immune response* because inside germinal centers, B cells proliferate expeditiously with the immunoglobulin variable region of the new B cells diversified by somatic hypermutation, resulting in the production of new generations of high affinity memory and plasma B cells^27^.

By default, *germinal center formation* (green GO term at the bottom of Fig. 2), is grouped in the *development* (under red dashed line) section rather than the *immune* section (under blue dotted line) due to it favoring the goslim GO header, *anatomical structure formation in morphogenesis* (red box). Section names for each GO are also indicated by the text at the bottom of each GO box.

The GO header, *anatomical structure formation in morphogenesis* (red box), is chosen from five possible GO headers (GO terms with a blue border) to represent *germinal center formation* because it has the smallest dcnt value (878) compared to 1796, 6004, 6473, and 29625. To move *germinal center formation* from *development* to *immune*, add a new GO header, *immune response*, (blue box) to the *immune* section. The GO term, *germinal center development*, will then be moved to the *immune* section because *immune response* has a dcnt of 835 which is less than 878.

Knowing that the research question concerns the role of the immune system in a particular condition and seeing numerous GOEA results in “immune”, a researcher may wish to guide a GO grouping of GOEA results such that a succinct summary clearly highlights the immune findings and the genes associated with those immune findings. Alternately, the researcher may prefer to highlight only the developmental aspect of germinal centers or both high-level descriptions, *developmental process* and *immune response*, at the cost of duplicating the GO term which makes the results list longer. Grouping is used to organize an already long list of GO terms to make the results easier to interpret, so making the list even longer may not be desired.

### GOATOOLS grouping compared to ReviGO visualization

GOATOOLS grouping can be preferable to tools such as ReviGO (Reduce and Visualize Gene Ontology)^28^ if the researcher wants to retain the full list of GO IDs returned from a GOEA, but organize the list so GO IDs are stored under large user-defined sections.

If a graphical visualization of the overall properties of all user GO IDs is desired, ReviGO is an excellent tool that can help visualize GO groupings using scatter plots, interactive graphs, and tag clouds. GOATOOLS grouping is list-based only. GOATOOLS GO plots are a tool for GO header placement decisions in the sections file and not considered a final output for an entire list of GO IDs.

ReviGO is desirable when the researcher wishes to reduce a list of GO terms using ReviGO’s redundancy reduction. GOATOOLS grouping philosophy is to retain the full list of GO IDs.

GOATOOLS grouping also allows the user to move groups of GO IDs from one section to another. This is necessary because GO terms can be correctly represented under more than one section. A researcher may wish to guide the specific section for the placement of the GO IDs using the research hypotheses and the GOEA results.

GOATOOLS grouping is preferable if the researcher wishes to retain the full list of statistically significant GO IDs, have control over choosing from multiple equally valid grouping decisions, and prefers to see the GO IDs in a list rather than a figure.

### Example usage of the Python API

An example of code which groups GO IDs into user-created sections is as follows, with many more code examples available as Jupyter notebooks on Github:

import collections as cx

from goatools.test_data.goatools_goea_consistent_increase import goea_results

from goatools.test_data.sections.gjoneska_pfenning import SECTIONS

from goatools.grouper.grouper import wr_xlsx_gos

**xlsx1 = "goids_consistent_increase.xlsx"**

**xlsx2 = "goids_consistent_increase_dcnt.xlsx"**

# NtGoeaResults = cx.namedtuple("NtGoeaResults", "GO p_fdr_bh name ...

# goea_results = [

# NtGoeaResults(GO=‘GO:0035458’, p_fdr_bh=4.21e-07, name=’cellular response to ...

# NtGoeaResults(GO=‘GO:0002376’, p_fdr_bh=4.32e-07, name=‘immune system process’,

# NtGoeaResults(GO=‘GO:0006954’, p_fdr_bh=4.74e-07, name=‘inflammatory response’,

# ...

**goids = [nt.GO for nt in goea_results if nt.p_fdr_bh < 0.05 and nt.enrichment == ‘e’]**

# SECTIONS = [# 18 sections

# ("immune", [# 15 GO-headers

# "GO:0002376", # immune system process

# "GO:0002682", # regulation of immune system process

# "GO:0030155", # regulation of cell adhesion

# ...

#]),

# ("viral/bacteria", [# 4 GO-headers

# "GO:0016032", # viral process

# "GO:0050792", # regulation of viral process

# "GO:0098542", # defense response to other organism

# ...

#]),

# ...

# GROUPING OPTION #1:

# The most specific GO header is determined using information content calculated using the annotations.

**wr_xlsx_gos(xlsx1, goids, sections=SECTIONS, gaf=**‘**gene_assocation.mgi’)**

# GROUPING OPTION #2:

# The most specific GO header is determined using descendants count.

**wr_xlsx_gos(xlsx2, goids, sections=SECTIONS)**

### Web implementation

The grouping functionality may be run from a Python script as shown above or from the website (http://goatools.org).

### Case study: The GP dataset

We used the GP gene expression data to compare the GOEA results among four different tools. The first tool was the older DAVID version 6.7 released in 2010^4^ and is referred to as “DAVID6.7”. The second was the most recent version of DAVID version 6.8, a major update completed in October 2016 and is referred to as “DAVID6.8”. The third set of GOEA results was generated by running GOstats, an extremely popular tool for running gene ontology analyses using the statistical language, R. The fourth set of GOEA results was generated by running GOATOOLS v0.8.2^29^.

#### Versions of ontologies, annotations and tools

We used the following versions of ontologies, annotations, and tools for the four utilities analyzed in this paper. First, our DAVID6.7 analyses use the DAVID Knowledgebase released Sep 2009 with version 6.7 of the DAVID software released Jan 2010. Second, our DAVID6.8 analyses use the DAVID Knowledgebase released May 2016 with version 6.8 of the DAVID software released Oct 2016. Third, our GOstats analyses use GO.db from NCBI gene Sep 21, 2016 and org.Mm.eg.db version 3.4.0 released Oct 2, 2016. The GOstats software used is in Bioconductor version 3.32 (Oct 31, 2016). Fourth, our GOATOOLS analyses use the ontologies in go-basic.obo released Apr 21, 2018 and annotations from gene_association.mgi released Apr 2, 2018. Finally, GOATOOLS grouping used GO slims from goslim_generic.obo downloaded Apr 22, 2018. All GOATOOLS analyses were run using GOATOOLS version 0.8.2 released Feb 22 2018.

To generate the DAVID6.7 GOEAs, we used the DAVID annotation set, GOTERM_BP_ALL, because it was used to generate the GOEA data found in GP’s Supplemental Table 2. Also, GOTERM_BP_ALL was the set of annotations available in DAVID which produced results closest to the GOATOOLS GOEA results.

To generate the DAVID6.8 GOEAs, we used the newly available GOTERM_BP_DIRECT annotations because it is the original unmodified annotations, which is what we used for all GOATOOLS analyses in this paper. The GOTERM_BP_ALL annotations augment the original annotations by propagating parent GO terms up the hierarchy.

For our cross-tool comparisons, we had to use two different sets of DAVID annotations because GOTERM_BP_DIRECT was not available in DAVID6.7. So we added a comparison of DAVID6.7 and DAVID6.8 using GOTERM_BP_ALL for both to examine the effect of using old versus new annotations.

#### The GP data set

Gjoneska and Pfenning’s gene expression data was used to investigated immunity in Alzheimer’s disease using mice that can be induced to display Alzheimer-like extreme neuronal loss and increased beta-amyloid peptide production and tau pathology^4^.

Gjoneska and Pfenning measured the gene expression of cells in the hippocampus at early (2 weeks after induction) and late stages (6 weeks after induction) after inducing the Alzheimer model. The Gjoneska gene expression results are organized into upregulated and downregulated genes at three time-points each. The first of the three time-points is *Transient* indicating the gene expression was only seen in the early stage (2 weeks). The second time point, *Consistent*, indicates the gene expression was seen in both early and late stages (both 2 and 6 weeks). The third time point, *Late*, indicates the gene expression was seen in only the late stage (6 weeks).

Gjoneska and Pfenning found an upregulation of immune genes and a downregulation of synaptic plasticity genes. We compare the upregulated immune results found in the Gjoneska paper using all four tools. The genes we examined are the upregulated genes in the three clusters; *Transient Increase* (TI), *Consistent Increase* (CI), and *Late Increase* (LI). Immune and viral or bacterial functions of statistical significance were the focus for our studies across the four tools. The population and study gene sets used in our GOEAs are from Gjoneska and Pfenning’s supplementary table one, *Gene expression differences in the CK-p25 mouse*.

## Results and Discussion

We compared GOEA results from GOATOOLS and the other tools through both stochastic simulations as well as real-world case study from Gjoneska *et al*. Overall, we show that GOATOOLS provides GO terms by median descendant count are twenty times more specific than the broad GO terms from DAVID6.7, two times more specific that GOstats, and similar in specificity to DAVID6.8 GO terms.

### Stochastic simulation study

**Figure 3:**
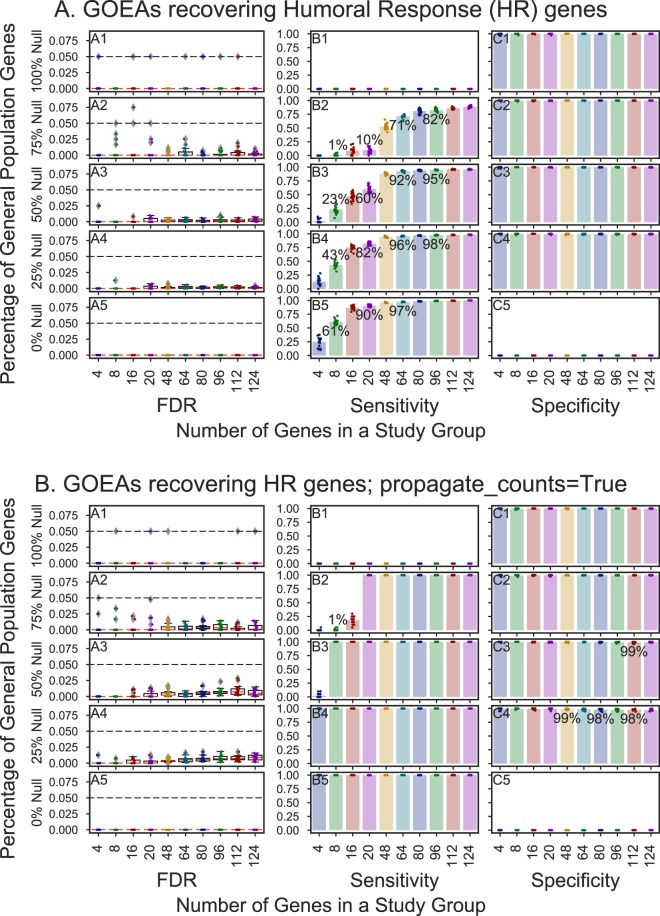
Results for 20,000 GOATOOLS GOEA stochastic simulations with varying sensitivity and consistently high specificity. GOEAs performed well on study groups of 8+ genes if the GOATOOLS GOEA option propagate_counts set to True.

GOATOOLS GOEA performance was tested by running 100,000 stochastic gene ontology enrichment analyses (GOEAs) simulations (Fig. 3 and Supplemental Figures S1–S3). Each simulation tested the correctly identified enrichment in a stochastically generated study gene list whose size ranged from four to 124 genes against a population of more than 20,000 mouse protein-coding genes. The study gene lists contained two types of randomly chosen genes: target genes and background genes^30^. Uncorrected P-values were generated using Fishers exact test. Corrected P-values were generated using Benjamini/Hochberg multiple test correction.

The target gene pool contains 124 genes associated with the humoral response (HR) biological process. The background gene pool contains the entire list of protein-coding genes excluding HR genes. One study set of genes contains one of the following percentages of background genes, also known as *Null* or *True Null* genes: 0%, 25%, 50%, 75%, and 100%. A study set of 100% null genes contains genes chosen only from the background set (Fig. 3, row 1). A study set of 0% null genes contains only randomly chosen HR genes (Fig. 3, row 5). A study set of 16 genes containing 25% null genes contains 4 randomly chosen background genes and 12 randomly chosen HR genes (Fig. 3, row 4). The target genes function as true positives in the GOEA while the background genes are counted as false positives.

### Simulation study results

The first simulations contained unacceptably high FDRs for larger study gene groups (Supplemental Figure S1). Upon investigation of the failing FDRs, there were two characteristics of GO IDs that were associated with the false positive study genes. First, the GO IDs related to the false positives are associated with thousands of genes. This is contrasted to the statistics for the overall mouse protein-coding associations: median = 3 genes/GO; mean = 16 genes/GO, and stddev = 128. Second, the GO IDs are much more likely to be under-represented, rather than enriched. An under-represented term is one in which far fewer genes appeared significant in the study set than in the general population.

Upon running the simulations viewing only *enriched* gene lists, the simulations solidly passed resulting in FDRs that were nearly zero (Fig. 3). Only 30 GO IDs out of over 17,000 GO IDs associated with mouse protein-coding genes are associated with over 1,000 genes. Upon running the simulations using an association with 30 GO IDs pruned out of the association, the simulations also passed with FDR values close to zero.

Performing stress tests by randomly shuffling the associations for True-Null genes prior to simulation, the “view-enriched-gene” simulations either passed or were very close to passing (Supplemental Figure S2) and all “30-GOs-Purged” simulations passed (Supplemental Figure S3).

The results of the GOATOOLS GOEA simulations show excellent FDR and specificity levels (Fig. 3A). The sensitivity varied with studies having 64+ genes performing well and study sizes of 4 genes performing poorly (Fig. 3A, panels B2 to B5), where truly enriched genes were not identified. Study sets containing 16 gene study sets performed well if 75%+ of the 16 study genes were truly enriched (Fig. 3A, panels B4 and B5). These simulation results are true only when viewing genes associated with statistically significant GO IDs that are enriched, not under-represented. Adding genes associated with under-represented GO terms resulted in an unacceptably high ratio (>0.05) of genes seen as associated with significant functions (Supplemental Fig. 1). The GOEA sensitivity is greatly improved, especially for small (4–20 genes) gene groups, if the option *propagate_counts* is set to “True,” which updates the annotations such that a gene’s associated GO terms now include all parent GO terms (Fig. 3B, panels B4 and B5). To compare results among the four tools, *propagate_counts* is set to the more conservative value, “False,” in GOATOOLS GOEAs which causes the annotations to be used in their original form with no modifications.

To recreate all five of our stochastic GOEA simulation plots (for a total of 100,000 total stochastic simulations) featured in the GOATOOLS manuscript and supplemental data, clone the repository, https://github.com/dvklopfenstein/goatools_simulation, and run this make target from the command line:

$ make run_ms

Generating the five simulation plots in the GOATOOLS manuscript and supplemental data takes about 38 hours on a laptop PC running an Intel(R) Core(TM) i7-6500U and 16 GB of RAM.

### Counts of genes associated with statistically significant GO terms

**Figure 4:**
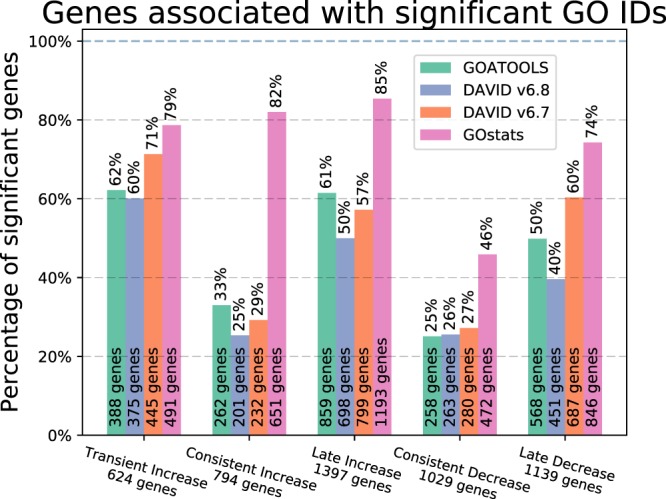
Percentages of genes associated with statistically significant GO terms for all tools and all GP clusters. The GOEA analyses from four different tools found significant GO IDs for five of the six GP gene clusters using the GP population background of 13,838 genes. The x-axis shows the five GP clusters and the total count of genes found to be up or down regulated in the GP experiments. The number of genes in each cluster that are found to be associated with significant GO IDs for each tool is printed at the bottom of each tool bar. The color of each bar represents a GOEA tool as specified in the legend. The height of each bar is the percentage of genes in each cluster that are found to be associated with significant GO IDs. GOATOOLS is most similar to the DAVID tools. GOstats found between 74% and 84% of the genes significant for four clusters, which will be reduced if the statistically significant but extremely broad term, *cellular process*, is removed.

GOATOOLS and DAVID6.7, and DAVID6.8 total gene counts for the GP study sets are much more similar (2,335, 2,443, and 1,988 respectively) than gene counts for GOstats (3,652 total genes) (Fig. 4). Looking closer, if we remove only *cellular process* (GO:0009987), an extremely broad depth-01 term with more than 18,000 GO term descendants out of a total of more than 29,000 in the entire *biological process* branch, the total genes associated with significant GO IDs reduce from 3,652 to 3,310 (342 genes removed) for GOstats (Supplemental Fig. 4). The genes that are removed are associated with *cellular process* and with no other GO IDs in the GOstats GOEA results. Removing *cellular process* has no effect on the GOATOOLS or DAVID6.8 results which do not show significance for *cellular process* although there are numerous more specific GO terms under *cellular process* that are statistically significant.

In practice, we might consider the large list of genes directly associated with *cellular process* from GOstats rarely useful. There are 57 GO terms in DAVID6.7 and 57 GO terms in GOstats which are both broad (meaning the descendants count is over 200) and have associations of more than 100 study genes. There are no such GO terms in GOATOOLS and only 3 in DAVID6.8. Therefore, it may be desirable to not include some of these broad terms in a GOEA summary.

### Broad vs. specific GO terms by grouping

The four tools together found a total of 833 GO terms statistically significant. We first grouped the GO terms into sections using the popular annotation-associated value, *information content* (*tinfo*), and then created a second grouping using the species and tool agnostic value, *descendants count* (*dcnt*).

The two grouping methods showed strong concordance with 810 GO IDs (97%) agreeing on section placement. There was disagreement in section placement for 23 GO IDs (2.76%). One example of disagreement was that the GO term, *transmission of nerve impulse*, was placed in the *neurological* section using *dcnt* and *signaling* using *tinfo*. A second example of disagreement was that the GO term, *trophoblast giant cell differentiation*, was placed in the *reproduction* section using *tinfo* and the *Misc*. (uncategorized) section using *dcnt*. The researcher may override any of these section placements by adding more specific GO headers to place the GO IDs of interest into a section which better informs the research question.

We chose to use *dcnt* rather than *tinfo* to compare GO results because *tinfo* values are determined using a set of annotations. But the four annotation sets differed among the four tools as revealed by the GO terms having different sets of genes in their associations. To avoid choosing annotations used by a single tool to evaluate all tools, we used *dcnt* to group the GO terms. In general *tinfo* may preferable to use in grouping decisions because it is determined by the annotations. But *dcnt* can be used if comparing sets of GO terms whose annotations differ, like when comparing sets of GO terms between different species or different tools.

**Figure 5:**
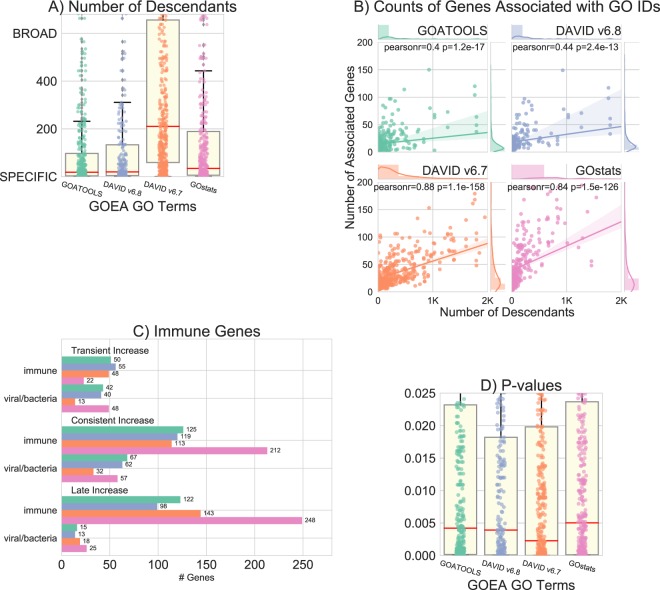
Comparison between enriched terms identified by GOATOOLS, DAVID and GOstats. All panels use the the same color coding as specified in the legend in Fig. 4. (**A**) Number of descendants for the significant terms reported in GOATOOLS, DAVID6.7, DAVID6.8, and GOstats. GOATOOLS and DAVID6.8 both discovered GO IDs with the lowest and most similar specificity. GOstats median GO ID broadness was twice that of GOATOOLS and DAVID6.8. DAVID6.7 discovered GO IDs 10x as broad as GOATOOLS when using mean *dcnt*. (**B**) Broader GO terms are associated with more genes while specific GO terms are associated with fewer genes. All four tools show a positive correlation between descendants count and the number of genes associated with the GO term. GOATOOLS and the most recent DAVID generally discovered very specific GO IDs associated with fewer genes. DAVID6.7 and GOstats found broader GO IDs that were associated with large numbers of genes. (**C**) Clusters and counts of genes significant for terms related to immunity. GOATOOLS and DAVID6.8 are most similar in the types and numbers of genes discovered. GOATOOLS discovered genes significant in immune and viral/bacterial categories for all GP clusters contrasted to the DAVID6.7 which found no viral/bacteria genes for any cluster. GOstats often found more genes, but they were often associated with broad GO IDs. (**D**) Comparison of P-values for all of the GO terms found in total in all four tools. The mean P-values were similar for GOATOOLS, DAVID6.8, and GOstats. DAVID6.7 had P-values multitudes lower than all the other tools.

In general, GOATOOLS GOEA GO ID results were consistently much closer to leaf-level, as measured by descendant count, than the DAVID6.7 results and the GOstats results (Fig. 5A). The GOstats results were much lower than the DAVID6.7 results with a median descendant count of 35 (mean = 491, SD = 2,198) for GOstats compared to a median of 211 (mean = 775, SD = 1,678) for DAVID6.7. GOATOOLS (median = 19, mean = 199, SD = 781) and DAVID6.8 descendants counts (median = 20, mean = 197, SD = 711) distributions were most similar among all tools examined. We saw a trend where GO terms closer to the bottom of the DAG, terms considered to be more specific, are associated with fewer genes (Fig. 5B).

### Example functional groups: immunity and viral/bacteria

Genes associated with statistically significant immune GO terms were found in all three upregulated clusters by all four tools (Fig. 5C).

To view the results of the GOEAs, we chose to split GO terms related to virus or other parasites into their own *viral/bacteria* section. Genes associated with *viral/bacteria* were found in all three clusters by all tools. GOATOOLS GOEAs found 42, 67, and 15 study genes with statistically significant GOs in the *viral/bacteria* section in all three clusters: *Transient*, *Consistent*, and *Late Increase*. GOstats found more genes than GOATOOLS (48, 57, and 25).

The GOATOOLS GOEAs found 50, 125, and 122 study genes associated with statistically significant GOs in the *immune* section in three clusters: *Transient*, *Consistent*, and *Late Increase*. DAVID6.8 found slightly fewer genes (55, 119, 98) than GOATOOLS. GOstats found generally more genes than GOATOOLS in the clusters (22, 212, 248).

GOATOOLS and DAVID6.8 reported similar number of associated genes no matter the level of the GO. GOATOOLS and DAVID6.7 reported different number of associated genes no matter the level of the GO. Curiously, *lymphocyte aggregation*, with a very low dcnt of 5 found significant by GOstats, but not by GOATOOLS was associated with 46 genes (Supplemental Table 3). Although it failed to reject the null hypothesis by GOATOOLS, *lymphocyte aggregation* was only associated with one gene in the association from MGI.

### Differences among tools

As an example of evaluating the differences between the results from the four tools, we describe the statistically significant GO terms in the *immune* section for the *Consistent Increase* cluster comparing GOATOOLS vs DAVID6.7 (Supplemental Table 1), DAVID6.8 (Supplemental Table 2), and GOstats (Supplemental Table 3). These tables are each sorted by descendant counts such that broader terms are listed before specific terms.

The DAVID6.7 terms tend to be concentrated at the top among the broader terms while missing specific GO terms at the bottom of the table that were found by GOATOOLS (Supplemental Table 1). GOATOOLS found 6 broader terms also found by DAVID6.7. But most terms found by GOATOOLS are extremely specific having a dcnt less than or equal to 11. For example, *positive regulation of interleukin-1 beta secretion*, with a depth of 11 in the third row from the bottom of the table is significant in the GOATOOLS GOEA and is associated with nine genes in the study. The statistically significant GO terms in the *immune* section are associated with a total of 125 genes as found by the GOATOOLS GOEA and 113 genes as found by the DAVID6.7 GOEA. The asterisk in most of the GOATOOLS P-value column indicates that where DAVID6.7 found a broader term significant, GOATOOLS found a more specific term in that term’s descendants significant.

DAVID6.8 performs much more similarly to GOATOOLS (Supplemental Table 2). When both GOATOOLS and DAVID6.8 find the same GO term, the number of associated genes is similar for the two tools indicating the associations used by the two tools are similar. GOATOOLS finds more GO terms significant than DAVID6.8. GOATOOLS finds GO terms that are more specific than found by DAVID6.8.

In the comparison between GOATOOLS and GOstats (Supplemental Table 3), the more specific bottom half of the table has similar GO term findings between the two tools. Additionally, in the bottom half of the table, when both GOATOOLS and GOstats find the same GO term, the number of associated genes is similar. The top half of the table showing the broader GO terms is where we see the larger differences between GOATOOLS and GOstats. The largest difference seen in the top half of the table is when GO terms are found by both tools, GOstats reports many more study genes associated with the GO term than reported by GOATOOLS.

### GO term overlaps among tools

The total counts of significant GO terms found by GOATOOLS, DAVID6.8, DAVID6.7, and GOstats is 383, 230, 390, and 428, respectively. GOATOOLS found the same GO IDs as DAVID6.8, DAVID6.7, and GOstats in the quantities of 227, 110, and 206. GOATOOLS and DAVID6.8 had the most concordance. GOATOOLS found hundreds of more specific GO terms than in DAVID6.7. But in DAVID6.8, the specificities of the GO terms were well-matched with those of GOATOOLS.

Examples of terms that are close to leaf-level found by GOATOOLS, but not found by DAVID6.8 or GOstats in the *Late Increase* cluster include *toll-like receptor signaling pathway*, *natural killer cell differentiation*, “*complement activation*, *classical pathway*,” and *neutrophil chemotaxis*. Both GOATOOLS and GOstats found *GO:0045576 mast cell activation* significant while DAVID6.8 did not.

Sometimes, one tool would find significance in a broader term that was not found by GOATOOLS. However, that broader term was actually covered by GOATOOLS by finding more specific children under the “missing” broader term. For example, “*antigen processing and presentation*” is found in GOstats and DAVID6.7 but not in GOATOOLS. But the more specific GO term under it, “*antigen processing and presentation of endogenous peptide antigen via MHC class I via ER pathway*, *TAP-dependent*,” was found statistically significant only in GOATOOLS. The counts of broader terms found by other tools that were actually covered by GOATOOLS finding more specific children terms are 166 for GOstats, 10 for DAVID6.8, and 291 for DAVID6.7.

**Figure 6:**
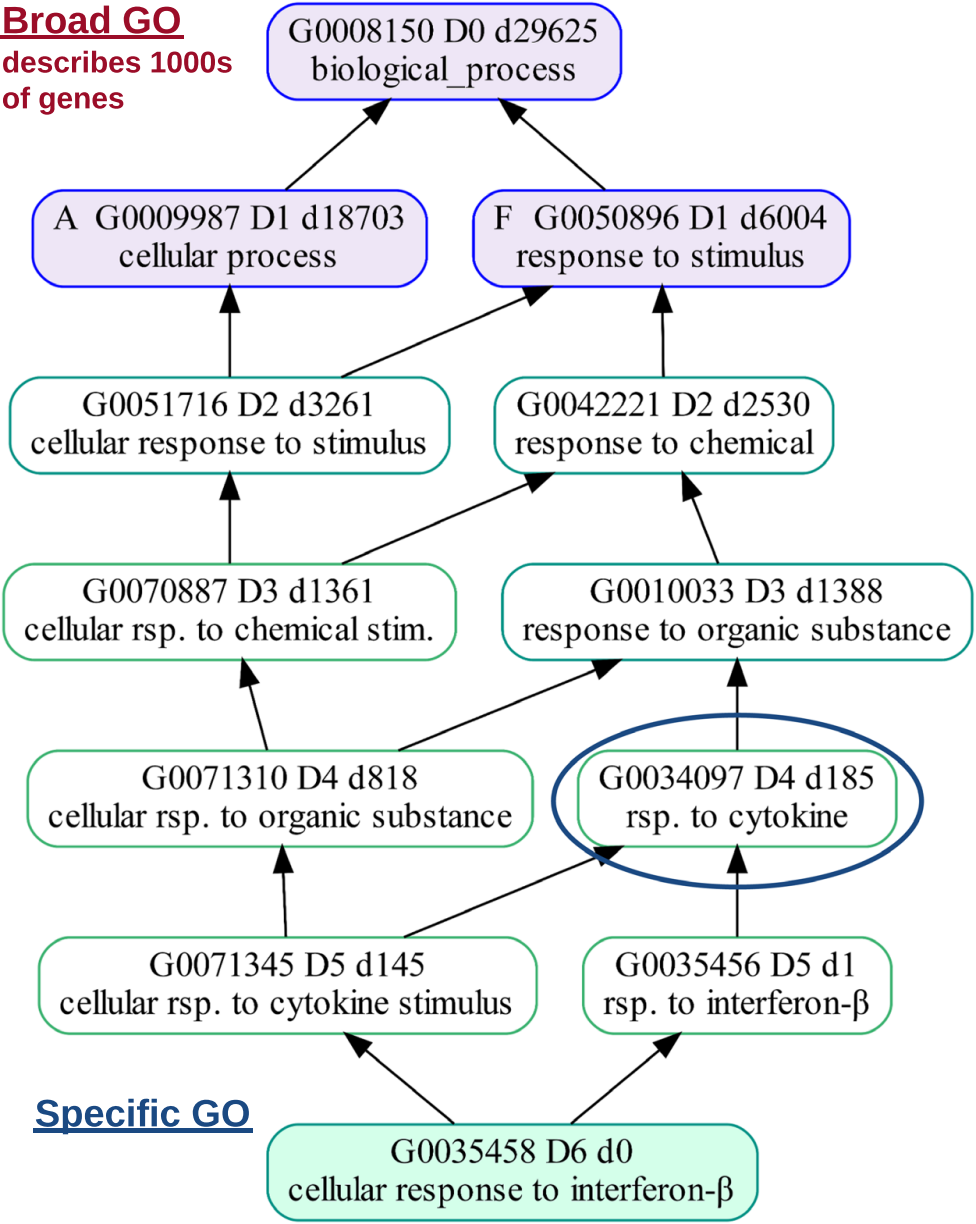
Leaf-level GO terms may initially have broad GO headers that do not convey enough information about a leaf-level term. The leaf-level GO term *cellular response to interferon-beta* (green) has three potential GO headers (top three GO terms with a blue border) that are extremely broad. Even *response to stimulus* is broad because its descendants are as diverse as *response to gravity*, *startle response*, and *immune response*. The user may wish to add a new GO header which better represents *cellular response to interferon-beta*, like *response to cytokine* (circled in blue). Colors in the GO term boxes indicate if one (purple) or more (green) tools found a GO term significant. GOATOOLS, DAVID6.8, and GOstats found *cellular response to interferon-beta* (green) significant. GOstats found the broad terms, *biological_process*, *cellular process*, and *response to stimulus* significant (purple). The blue GO term borders indicate that a GO term is also a GO header. Of the GO terms pictured here, only *biological_process* is found in the GO slims as of April 2018. The depth-01 GO terms are default header GO terms because GOATOOLS grouping adds all depth-01 GO terms to the list of default headers.

At times, broad GO terms with low information content (i.e. associated with large quantities of gene products) found in GOEAs may not meaningfully map to the more specific GO terms. For example, GOstats found *cellular process* significant (purple, on left) in Fig. 6. The purple terms are terms found significant in GOstats but not found significant in either DAVID6.8 or GOATOOLS. A specific GO term that could be represented by *cellular process* is the leaf-level term *cellular response to interferon-beta* (green), which is found significant in GOATOOLS, GOstats, and DAVID6.8. The purple GO header terms are so broad that we cannot be sure that they meaningfully cover the specific GO term, *cellular response to interferon-beta* (green, bottom). Even *response to stimulus* (purple, top right) is an extremely broad umbrella term encompassing terms as diverse as *eye blink reflex* and *innate immune response* and is not a meaningful proxy to represent *cellular response to interferon-beta*.

### Summary

GOATOOLS results were most similar to DAVID6.8′s results when using DAVID’s new GOTERM_BP_DIRECT GO set in terms of numbers of genes found, the P-value values, and the similarity of the GO term specificity. GOstats and DAVID6.7 found more broad terms, but that is likely because they employ some form of propagate counts to augment the original association. DAVID6.7 misses many specific terms.

#### Using the latest ontologies and annotations

The main difference between the GOATOOLS GOEA results and the DAVID6.8 results was that GOATOOLS found specific GO terms not found by DAVID6.8. This could be a consequence of being able to use the very latest ontologies and annotations in GOATOOLS, a crucial factor that influences all GO term enrichment results and is described in a recent article by Wadi *et al*., in Nature Methods^31^. Wadi reports that using old annotation datasets and old ontology versions has influenced the results of thousands of recent studies by markedly underestimating the functional significance of their gene lists, negatively affecting follow-up studies.

Both the ontologies and the annotations change daily, with the number of human biological processes doubling from 6,509 in 2009 to 14,735 in 2016^31^. In the ontologies, the GO vocabulary is increasingly expanded, resulting with GO terms having longer paths to roots and terms having more parents. Some GO terms are rendered obsolete and are pruned as biological knowledge expands. The number and quality of annotations per gene constantly increases with high-confidence experimental annotations becoming more frequent, and more genes annotated. Poor quality annotations are pruned due to constant quality control efforts. Annotations can vary among tools.

That GOATOOLS and DAVID6.8 performed with greater similarity than DAVID6.7 could be due to the fairly recent update to DAVID.

One of the greatest benefits of using GOATOOLS is that the researcher has full control of the versions of the GO and the annotations that he or she uses. The full GOEA project can be archived including the ontology, annotation, study and population gene product sets, and code used to generate the GOEA results since all text files and code and accessible to the researcher.

#### Effects of old and new annotations in DAVID

To get a sense of how the dates of the GO DAG and the annotations might affect GOEA results, we compared DAVID6.7 and DAVID6.8 GOEAs whose Benjamini values were less than 0.05 using DAVID’s GOTERM_BP_ALL terms for both sets of analyses.

The most notable difference was DAVID6.8 found about four times as many unique GO terms to be significant than were found by DAVID6.7 (1,617 GO terms for DAVID6.8 vs 390 GO terms for DAVID6.7) for all GP clusters.

Also using the GO DAG downloaded in April 2018, the dcnt for significant GO IDs for DAVID6.8 was smaller (52 median, 296 mean, SD = 1,192) than for DAVID6.7 (163 median, 668 mean, SD = 1,672).

#### Effects of the same annotations in DAVID6.8 and GOATOOLS

To examine the effects of using the same annotations in DAVID and GOATOOLS, we ran GOATOOLS GOEAs with annotations downloaded from DAVID6.8 using *Fisher*’*s exact test* and both the *Benjamini-Hochberg* and *Bonferroni* multiple test corrections. GOATOOLS generally found more GO terms than found by DAVID6.8 (Supplemental Table 4).

#### Tool comparison

The P-values found by all tools had similar statistics overall (Fig. 5D). DAVID6.7 found GO terms that were ten times broader than other tools and completely missed many specific GO terms (Fig. 5A). GOstats found GO terms that were almost twice as broad as GOATOOLS and DAVID6.8.

The broad GO terms found by GOstats and DAVID6.7 could sometimes be exceptionally broad and associated with hundreds of genes yielding impractical results. For example, the particularly broad depth-01 term, *cellular process* (GO:0009987) with its over 18,000 descendant GO terms may not be helpful in describing unique properties of a gene set. Also, such broad terms may result in the addition of discovering hundreds of genes that are only associated with broad terms having low information content (Supplemental Fig. 4). And finally, including extremely broad GO terms in GOEAs may cause GOEAs to have unacceptably high FDRs which exceed the alpha set by the researcher (Supplemental Fig. 1).

GOstats and DAVID6.7 (using the GOTERM_BP_ALL GO set) found more broader GO terms than GOATOOLS and DAVID6.8 (using the new GOTERM_BP_DIRECT GO set). Finding more broader GO terms may be due to GOstats and DAVID6.7 using a variation of propagate_counts to augment the original annotations.

Our stochastic simulations show that using propagate_counts can result in greater sensitivity to find truly enriched genes (3B) rather than missing them. If using propagate_counts, it may be especially important to remove extremely broad GO terms that are better represented by numerous specific GO terms prior to the analyses to prevent FDR values from exceeding the alpha set by the researcher.

We chose to not use propagate_counts in GOATOOLS and to use the GOTERM_BP_DIRECT DAVID annotation set for the analyses in this paper to investigate the GOEA results using original unmodified annotations. In actual practice, it may be desirable to run GOEAs trying both the original unmodified annotations and propagate_counts.

## Conclusion

The first stochastic simulations failed, meaning that the FDR exceeded the alpha set by the researcher (Supplemental Fig. 1). The source of the failures were false positives involving extremely broad GO terms associated with more than one thousand genes for *biological_process* in the mouse annotations. Simulations passed if just 30 broad GO terms out of more than 17,000 total annotated GO terms are removed from the annotations prior to running the GOEAs. Therefore, developers of GOEA tools may want to consider removing even a small number of GO IDs associated with large numbers of genes if the broad GO term may be better represented by numerous annotated more specific descendant GO terms.

Stochastic simulations revealed that augmenting the annotations using *propagate_count* set to “True” to cause parent GO terms to be added to a gene product’s annotations resulted in better sensitivity in finding truly enriched results which would otherwise not be found (Fig. 3B). Smaller study gene sets (4–20 gene products) most dramatically benefited from propagating GO annotations depending on the percentage of truly enriched gene products in the study sets (Fig. 3B, panels B3–B5).

Because using any variation of *propagate_counts* comes at the expense of finding more broad terms, developers of GOEA tools should strongly consider pruning selected broad terms that are associated with large numbers of genes and have numerous descendants prior to running GOEAs. Researchers may wish to run a GOEA twice, once with the original annotations and once with the annotations augmented by propagating annotations up through GO parents.

Numerous GO terms, especially large groups of specific GO terms, can be difficult to summarize. GOATOOLS grouping not only makes a single set of GOEA results easier to understand from a systems level, but it also makes it possible to compare GOEA results across multiple tools, species, or experiments even if the GO terms from the various tools or experiments are at different depths.

The GOATOOLS library can help the researcher keep current with rapidly changing ontologies and associations as well as organize and summarize GOEA results. Given Python’s popularity among bioinformaticians and data scientists, GOATOOLS fills a significant void while maintaining comparable if not better performance than other tools and libraries that are built using other programming languages.

## Electronic supplementary material

Supplementary Information
