# Supplementary material for: GOATOOLS: A Python library for Gene Ontology analyses
Source: Sci Rep. 2018 Jul 18;8:10872. doi: 10.1038/s41598-018-28948-z (PMC6052049; doi:10.1038/s41598-018-28948-z)
Supplement: Supplementary file 1 — Supplementary Information [file 41598_2018_28948_MOESM1_ESM.pdf]

GOATOOLS: A Python library for Gene Ontology analyses

Supplemental Material

D. V. Klopfenstein, Liangsheng Zhang, Brent S. Pedersen, Fidel Ramírez, Alex Warwick Vesztrocy, Aurélien Naldi, Christopher J. Mungall, Jeffrey M. Yunes, Olga Botvinnik, Mark Weigel, Will Dampier, Christophe Dessimoz, Patrick Flick, and Haibao Tang

Supplemental Tables

Immune Summary in the *Consistent Increase* Gjoneska cluster with DAVID version 6.7 released Jan 2010

dcnt	info	D	GO	Description	GOATOOL	DAVID6.7	/125	/113
1796	6.4	1	GO:0002376	immune system process	5.28e-07	7.96e-20	64	95
1128	6.6	3	GO:0002682	regulation of immune system process	*	3.03e-08	0	41
835	6.9	2	GO:0006955	immune response	7.84e-04	2.12e-17	18	62
596	7.2	4	GO:0050776	regulation of immune response	*	1.19e-07	0	29
523	7.0	4	GO:0002684	+ reg. of immune sys. process	*	7.65e-07	0	29
502	7.6	2	GO:0002252	immune effector process	*	6.12e-05	0	21
495	12.2	4	GO:0002520	immune system development	*	1.20e-02	0	28
464	7.4	4	GO:0001817	regulation of cytokine production	7.84e-04	1.71e-03	8	19
443	7.8	3	GO:0045321	leukocyte activation		1.02e-02	0	23
352	8.5	4	GO:0048534	hematopoietic or lymphoid organ devel.	*	1.10e-02	0	27
334	7.5	5	GO:0050778	+ reg. of immune response	*	2.47e-07	0	24
333	8.0	4	GO:0002697	regulation of immune effector process	*	2.05e-03	0	14
333	9.1	5	GO:0030097	hemopoiesis	*	3.44e-02	0	24
288	7.5	4	GO:0045087	innate immune response	3.50e-07	4.73e-02	51	13
256	9.3	3	GO:0002443	leukocyte mediated immunity	*	2.28e-03	0	14
247	7.4	5	GO:0002694	regulation of leukocyte activation		1.33e-02	0	16
226	8.5	3	GO:0002250	adaptive immune response	4.36e-03	9.48e-03	13	13
211	9.4	4	GO:0002460	adaptive immune rsp. based on somatic recombin. of immune rx. built w/Ig domains	*	9.48e-03	0	13
205	7.6	6	GO:0051249	regulation of lymphocyte activation		4.28e-02	0	14
189	7.9	4	GO:0006954	inflammatory response	5.09e-07	7.58e-07	41	27
183	9.6	4	GO:0002449	lymphocyte mediated immunity	*	3.42e-02	0	11
179	7.9	6	GO:0002253	activation of immune response		2.90e-03	0	13
175	8.6	5	GO:0002703	regulation of leukocyte mediated immunity	*	5.02e-04	0	13
162	7.9	5	GO:0001819	+ reg. of cytokine production	*	7.36e-05	0	15
159	8.1	5	GO:0002764	immune response-regulating signaling pw.		8.90e-03	0	10
149	8.9	3	GO:0002263	cell activation in immune response		1.74e-02	0	8
146	8.8	5	GO:0002819	regulation of adaptive immune response	*	6.60e-05	0	13
144	8.9	4	GO:0002366	leukocyte activation in immune response		1.74e-02	0	8
143	8.1	7	GO:0002757	immune rsp-activating signal transduction		8.90e-03	0	10
136	8.8	6	GO:0002822	reg. of adaptive immune rsp. based on somatic recombin. of immune rx. built w/Ig superfamily domains	*	6.60e-05	0	13
134	8.2	7	GO:0050863	regulation of T cell activation		3.21e-02	0	12
128	9.7	2	GO:0019882	antigen processing and presentation	*	2.61e-08	0	20
125	8.8	6	GO:0002706	reg. of lymphocyte mediated immunity	*	8.72e-04	0	12
120	8.5	5	GO:0002699	positive reg. of immune effector process	*	8.36e-04	0	11
68	9.2	4	GO:0002274	myeloid leukocyte activation		1.51e-02	0	9
61	9.0	6	GO:0002705	pos. reg. of leukocyte mediated immunity	*	6.13e-04	0	10
58	10.3	3	GO:0048002	antigen processing/present of peptide Ag	*	4.17e-06	0	14
51	9.2	6	GO:0002821	pos. reg. of adaptive immune response	*	4.03e-04	0	10
48	9.2	7	GO:0002824	+reg. of adaptive immune rsp based on somatic recombin. of immune rx. built w/Ig superfamily domains	*	4.03e-04	0	10
44	9.2	7	GO:0002708	+reg. of lymphocyte mediated immunity	*	6.13e-04	0	10
40	8.3	6	GO:0002768	immune rsp.-reg. cell surface rx. sig. pw.		4.18e-02	0	8
37	9.7	7	GO:0002709	regulation of T cell mediated immunity	*	2.94e-03	0	8
28	8.3	8	GO:0002429	immune rsp.-activate cell surface rx sig. pw.		4.18e-02	0	8
17	11.1	4	GO:0002474	a.p.p. of peptide antigen via MHC class I	*	4.95e-04	0	9
16	9.9	5	GO:0001912	+reg. of leukocyte mediated cytotoxicity	*	2.23e-02	0	7

Copyright (C) 2009-2018. DV Klopfenstein, H Tang, et al. All rights reserved.

13	10.2	8	GO:0002711	pos. reg. of T cell mediated immunity	*	9.07e-04	0	8
11	11.8	4	GO:0032611	interleukin-1β production	2.06e-03		5	0
10	10.3	6	GO:0030316	osteoclast differentiation	3.51e-02		8	0
9	10.5	3	GO:0019884	a.p.p. of exogenous antigen	4.39e-02	6.77e-05	3	12
9	10.6	8	GO:0001914	regulation of T cell mediated cytotoxicity	*	6.27e-03	0	7
8	8.9	6	GO:0032680	regulation of TNF production	*	6.27e-03	0	8
8	9.1	5	GO:0034341	response to interferon-γ	1.56e-02		6	0
8	10.0	5	GO:0032653	regulation of interleukin-10 production		3.53e-02	0	4
7	9.0	5	GO:0032675	regulation of interleukin-6 production	*	4.93e-02	0	7
7	10.8	4	GO:0002478	a.p.p. of exogenous peptide antigen	*	9.06e-04	0	9
3	10.8	9	GO:0001916	+ reg. of cytotoxicity via T cell	5.40e-03	3.16e-03	6	7
2	9.4	6	GO:0032755	+ reg. of interleukin-6 production	3.81e-04	7.26e-03	12	7
2	9.4	7	GO:0032760	+ reg. of TNF production	7.91e-05	4.80e-03	14	7
2	9.6	6	GO:0032729	+ reg. of interferon-γ production	4.00e-02		7	0
2	11.2	9	GO:0002726	+ reg. of T cell cytokine production	3.87e-02		4	0
2	11.9	5	GO:0042590	a.p.p. of peptide antigen w/MHC class I	1.67e-02		4	0
1	11.3	9	GO:0045651	+ reg. of macrophage differentiation	3.44e-02		5	0
0	10.6	11	GO:0050718	+ reg. of interleukin-1β secretion	1.94e-04		9	0
0	11.2	5	GO:0019886	a.p.p. of peptide antigen w/MHC class II	4.74e-02		5	0
0	12.8	7	GO:0002485	a.p.p. of endogenous peptide antigen w/MHC-I w/ER pathway, TAP-dependent	4.39e-02		3	0

Table 1. GOATOOLS compared to the older DAVID6.7 for the Gjoneska *Consistent Increase* cluster for immune GO terms. GOATOOLS finds more specific GO terms than the older version of DAVID. Specific GO terms are at the bottom of the table. Broad GO terms are at the top of the table. An asterisk (”*”) in the GOATOOL P-value column means that although the GO term was not found statistically significant by GOATOOLS, a more specific descendant term was found statistically significant by GOATOOLS. The numbers in the dcnt column are descendant counts as counted using both the *is_a* attribute and relationships like *part_of* and *regulates*. The info column contains *information content* using the MGI annotations. The numbers under the column ”/125” are counts of Gjoneska genes associated with GO terms found significant by GOATOOLS. The numbers under the ”/113” column are counts of Gjoneska genes associated with GO terms found significant by DAVID6.7. The total amount of genes related to significant immune GO terms in the Gjoneska *Consistent Increase* is 125 for GOATOOLS and 113 for DAVID6.7.

Copyright (C) 2009-2018. DV Klopfenstein, H Tang, et al. All rights reserved.

Immune Summary in the *Consistent Increase* Gjoneska cluster with DAVID version 6.8 released Oct 2016

dcnt	info	D	GO	Description	GOATOOL	DAVID6.8	/125	/119
1796	6.4	1	GO:0002376	immune system process	5.28e-07	2.40e-18	64	61
835	6.9	2	GO:0006955	immune response	7.84e-04	4.75e-04	18	21
464	7.4	4	GO:0001817	regulation of cytokine production	7.84e-04	*	8	0
288	7.5	4	GO:0045087	innate immune response	3.50e-07	2.83e-12	51	49
226	8.5	3	GO:0002250	adaptive immune response	4.36e-03	3.90e-03	13	14
189	7.9	4	GO:0006954	inflammatory response	5.09e-07	2.95e-09	41	39
17	11.1	4	GO:0002474	a.p.p. of peptide antigen via MHC class I	*	5.16e-04	0	9
11	11.8	4	GO:0032611	interleukin-1β production	2.06e-03	2.20e-02	5	5
10	10.3	6	GO:0030316	osteoclast differentiation	3.51e-02		8	0
9	10.5	3	GO:0019884	a.p.p. of exogenous antigen	4.39e-02		3	0
8	9.1	5	GO:0034341	response to interferon-γ	1.56e-02	2.01e-02	6	7
4	9.3	6	GO:0071346	cellular response to interferon-γ		1.81e-03	0	11
3	10.8	9	GO:0001916	+ reg. of cytotoxicity via T cell	5.40e-03	3.70e-03	6	7
2	9.4	6	GO:0032755	+ reg. of interleukin-6 production	3.81e-04	6.86e-04	12	12
2	9.4	7	GO:0032760	+ reg. of TNF production	7.91e-05	1.73e-04	14	14
2	9.6	6	GO:0032729	+ reg. of interferon-γ production	4.00e-02		7	0
2	11.2	9	GO:0002726	+ reg. of T cell cytokine production	3.87e-02		4	0
2	11.9	5	GO:0042590	a.p.p. of peptide antigen w/MHC class I	1.67e-02		4	0
1	11.3	9	GO:0045651	+ reg. of macrophage differentiation	3.44e-02		5	0
0	10.6	11	GO:0050718	+ reg. of interleukin-1β secretion	1.94e-04	9.09e-03	9	7
0	11.2	5	GO:0019886	a.p.p. of peptide antigen w/MHC class II	4.74e-02		5	0
0	12.8	7	GO:0002485	a.p.p. of endogenous peptide antigen w/MHC class I w/ER pathway, TAP-dependent	4.39e-02		3	0

Table 2. GOATOOLS compared to the current DAVID6.8 for the Gjoneska *Consistent Increase* cluster for immune GO terms. GOATOOLS finds more specific GO terms than DAVID6.8. Specific GO terms are at the bottom of the table. Broad GO terms are at the top of the table. An asterisk ("*") in the GOATOOL or DAVID6.8 P-value columns means that although the GO term was not found statistically significant, a more specific descendant term was found statistically significant. The numbers in the dcnt column are descendant counts as counted using both the *is_a* attribute and relationships like *part_of* and *regulates*. The info column contains *information content* using the MGI annotations. The numbers under the column "/125" are counts of Gjoneska genes associated with GO terms found significant by GOATOOLS. The numbers under the "/119" column are counts of Gjoneska genes associated with GO terms found significant by DAVID6.8. The total amount of genes related to significant immune GO terms in the Gjoneska *Consistent Increase* is 125 for GOATOOLS and 119 for DAVID6.8

Copyright (C) 2009-2018. DV Klopfenstein, H Tang, et al. All rights reserved.

Immune Summary in the *Consistent Increase* Gjoneska cluster with GOstats from Sep 2016

dcnt	info	D	GO	Description	GOATOOL	GOstats	/125	/212
1796	6.4	1	GO:0002376	immune system process	5.28e-07	*	64	0
835	6.9	2	GO:0006955	immune response	7.84e-04	2.49e-02	18	126
495	12.2	4	GO:0002520	immune system development	*	1.84e-06	0	75
464	7.4	4	GO:0001817	regulation of cytokine production	7.84e-04	*	8	0
443	7.8	3	GO:0045321	leukocyte activation		4.68e-03	0	72
288	7.5	4	GO:0045087	innate immune response	3.50e-07	3.15e-08	51	72
247	7.4	5	GO:0002694	regulation of leukocyte activation		3.86e-02	0	43
226	8.5	3	GO:0002250	adaptive immune response	4.36e-03	5.68e-05	13	45
189	7.9	4	GO:0006954	inflammatory response	5.09e-07	3.20e-06	41	60
179	7.9	6	GO:0002253	activation of immune response		1.14e-03	0	35
166	7.9	5	GO:1903706	regulation of hemopoiesis	*	3.60e-03	0	39
159	8.1	5	GO:0002764	immune rsp-regulating signaling pathway		1.16e-04	0	29
149	8.9	3	GO:0002263	cell activation in immune response		2.25e-03	0	23
128	9.7	2	GO:0019882	antigen processing and presentation	*	3.86e-02	0	24
82	10.9	4	GO:0002444	myeloid leukocyte mediated immunity		3.71e-02	0	11
76	8.7	6	GO:0002695	negative regulation of leukocyte activation		4.19e-02	0	16
75	10.6	5	GO:0071887	leukocyte apoptotic process		3.24e-02	0	18
56	8.4	5	GO:0070663	regulation of leukocyte proliferation		2.79e-02	0	18
48	9.2	7	GO:0002824	+reg. of somatic recomb. of Ig immune rx.	*	1.58e-06	0	16
47	9.6	4	GO:0001910	reg. of leukocyte mediated cytotoxicity	*	1.16e-02	0	10
44	9.2	7	GO:0002708	+reg. of lymphocyte mediated immunity	*	1.84e-06	0	17
41	8.8	7	GO:1902107	+reg. of leukocyte differentiation	*	9.69e-05	0	20
38	9.9	9	GO:2000514	regulation of CD4+, α-β T cell activation		5.73e-03	0	8
37	9.7	7	GO:0002709	regulation of T cell mediated immunity	*	5.68e-05	0	12
28	8.3	8	GO:0002429	immune rsp.-activ. cell surface rx. sig. pw.		1.69e-02	0	18
26	8.9	5	GO:1903555	reg. of TNF superfamily cytokine prod.	*	2.55e-04	0	18
17	11.1	4	GO:0002474	a.p.p. of peptide antigen via MHC class I	*	7.88e-04	0	15
15	11.4	3	GO:0019883	a.p.p. of endogenous antigen	*	2.25e-03	0	7
11	11.8	4	GO:0032611	interleukin-1β production	2.06e-03	*	5	0
10	10.3	6	GO:0030316	osteoclast differentiation	3.51e-02		8	0
9	10.5	3	GO:0019884	a.p.p. of exogenous antigen	4.39e-02	*	3	0
8	9.1	5	GO:0034341	response to interferon-γ	1.56e-02	*	6	0
8	10.3	9	GO:0050704	regulation of interleukin-1 secretion	*	2.25e-03	0	7
7	11.9	5	GO:0019885	a.p.p. of endog. peptide antigen via MHC-I	*	5.14e-03	0	6
5	12.5	6	GO:0071593	lymphocyte aggregation		7.70e-08	0	46
4	9.3	6	GO:0071346	cellular response to interferon-γ		3.85e-02	0	8
3	10.8	9	GO:0001916	+ reg. of cytotoxicity via T cell	5.40e-03	2.22e-04	6	8
2	9.4	6	GO:0032755	+ reg. of interleukin-6 production	3.81e-04	8.45e-04	12	12
2	9.4	7	GO:0032760	+ reg. of TNF production	7.91e-05	1.37e-04	14	15
2	9.6	6	GO:0032729	+ reg. of interferon-γ production	4.00e-02	3.85e-02	7	9
2	10.1	6	GO:0032757	+ reg. of interleukin-8 production		1.93e-02	0	8
2	10.4	6	GO:0032733	+ reg. of interleukin-10 production		3.86e-02	0	7
2	11.2	9	GO:0002726	+ reg. of T cell cytokine production	3.87e-02	2.16e-02	4	5
2	11.9	5	GO:0042590	a.p.p. of peptide antigen w/MHC class I	1.67e-02	2.16e-02	4	4
1	11.3	9	GO:0045651	+ reg. of macrophage differentiation	3.44e-02	1.97e-02	5	5
0	10.6	11	GO:0050718	+ reg. of interleukin-1β secretion	1.94e-04	8.04e-04	9	7
0	11.2	5	GO:0019886	a.p.p. of peptide antigen w/MHC class II	4.74e-02	2.88e-02	5	5
0	12.8	7	GO:0002485	a.p.p. of endog. peptide antigen w/MHC-I w/ER pw., TAP-dependent	4.39e-02		3	0

Table 3. GOATOOLS compared to GOstats. Specific GO terms found by both GOATOOLS and GOstats had similar number of associated genes. Broad GO terms found by both showed GOstats was associated with more genes than GOATOOLS, indicating that the original annotations were augmented by propagating counts. See captions in S1 or S2 for table column descriptions.

Copyright (C) 2009-2018. DV Klopfenstein, H Tang, et al. All rights reserved.

Method	Category	GOATOOLS	DAV6.8	GD	G	D	Gjoneska Cluster
fdr_bh	GOTERM_BP_DIRECT	124	100	100	24	0	Transient Inc.
fdr_bh	GOTERM_BP_DIRECT	48	29	29	19	0	Consistent Inc.
fdr_bh	GOTERM_BP_DIRECT	135	53	53	82	0	Late Inc.
fdr_bh	GOTERM_BP_DIRECT	21	14	14	7	0	Consistent Dec.
fdr_bh	GOTERM_BP_DIRECT	69	54	54	15	0	Late Dec.
fdr_bh	GOTERM_BP_ALL	598	399	399	199	0	Transient Inc.
fdr_bh	GOTERM_BP_ALL	515	334	334	181	0	Consistent Inc.
fdr_bh	GOTERM_BP_ALL	1274	760	758	516	2	Late Inc.
fdr_bh	GOTERM_BP_ALL	215	178	167	48	11	Consistent Dec.
fdr_bh	GOTERM_BP_ALL	560	449	448	112	1	Late Dec.
bonferroni	GOTERM_BP_DIRECT	67	44	44	23	0	Transient Inc.
bonferroni	GOTERM_BP_DIRECT	25	16	16	9	0	Consistent Inc.
bonferroni	GOTERM_BP_DIRECT	29	14	14	15	0	Late Inc.
bonferroni	GOTERM_BP_DIRECT	5	5	5	0	0	Consistent Dec.
bonferroni	GOTERM_BP_DIRECT	31	22	22	9	0	Late Dec.
bonferroni	GOTERM_BP_ALL	270	227	227	43	0	Transient Inc.
bonferroni	GOTERM_BP_ALL	169	121	121	48	0	Consistent Inc.
bonferroni	GOTERM_BP_ALL	403	268	267	136	1	Late Inc.
bonferroni	GOTERM_BP_ALL	114	103	99	15	4	Consistent Dec.
bonferroni	GOTERM_BP_ALL	252	216	216	36	0	Late Dec.

Table 4. GOATOOLS found more statistically significant GO terms than found by DAVID6.8 when using the same annotations. The annotations used in the GOATOOLS GOEAs shown here were downloaded from DAVID6.8. GOATOOLS ran *Fisher's exact test* and both the *Bonferroni* and *Benjamini-Hochberg* multiple test corrections. Annotation sets downloaded from DAVID6.8 include GOTERM_BP_DIRECT and GOTERM_BP_ALL. GOTERM_BP_DIRECT is new in DAVID6.8 and provides GO mappings directly annotated by the source database. GOTERM_BP_ALL augments the original annotations by propagating parent GO terms up the hierarchy. GOATOOLS' Benjamini-Hochberg scores are compared against DAVID6.8's Benjamini scores. GOATOOLS' Bonferroni scores are compared against DAVID6.8's Bonferroni scores. The number of GO terms found statistically enriched (alpha=0.05) for GOATOOLS and DAVID6.8 is under column headers *GOATOOLS* and *DAV6.8*. The number of GO terms found enriched by both GOATOOLS and DAVID6.8 is listed in the *GD* column. The number of GO terms found only by GOATOOLS is listed in the *G* column. The number of GO terms found only by DAVID6.8 is listed in the *D* column. The last column contains Gjoneska clusters of genes found up-regulated (Inc. for Increase) or down-regulated (Dec. for Decrease) across three different time periods (Transient, Consistent, or Late).

Copyright (C) 2009-2018. DV Klopfenstein, H Tang, et al. All rights reserved.

Supplemental Figures

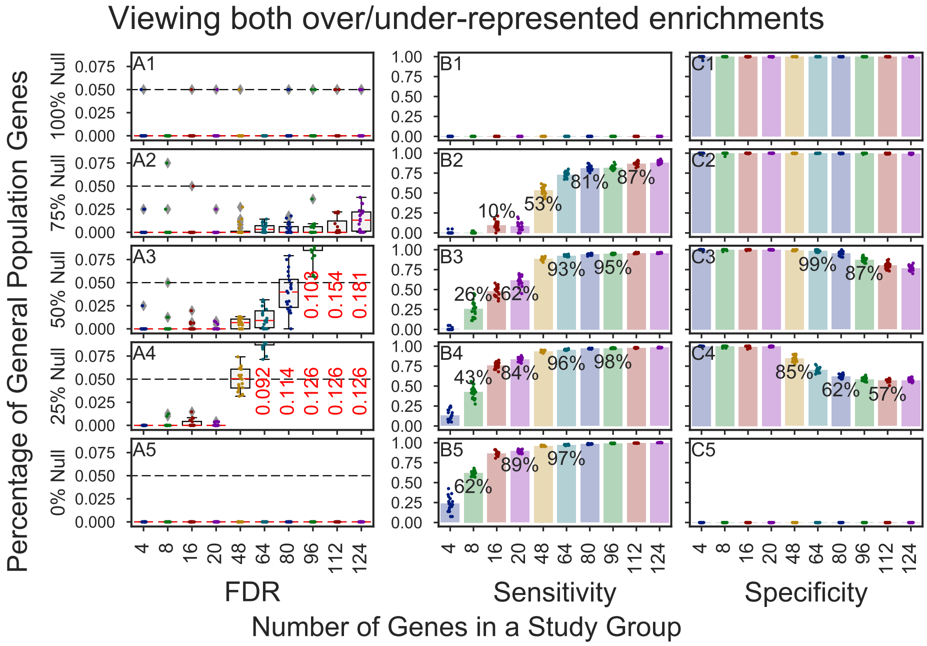

Figure 1. **The first GOATOOLS GOEA simulations fail in panels A3 and A4 with FDR values exceeding the alpha of 0.05 set by the researcher.** The values of failing FDRs are shown using red text. The source of the failures were false positives for GO terms annotated with large numbers of gene products. For mouse annotations in the *biological_process* branch, GO terms annotated with 1,000 or more genes were the source of failures.

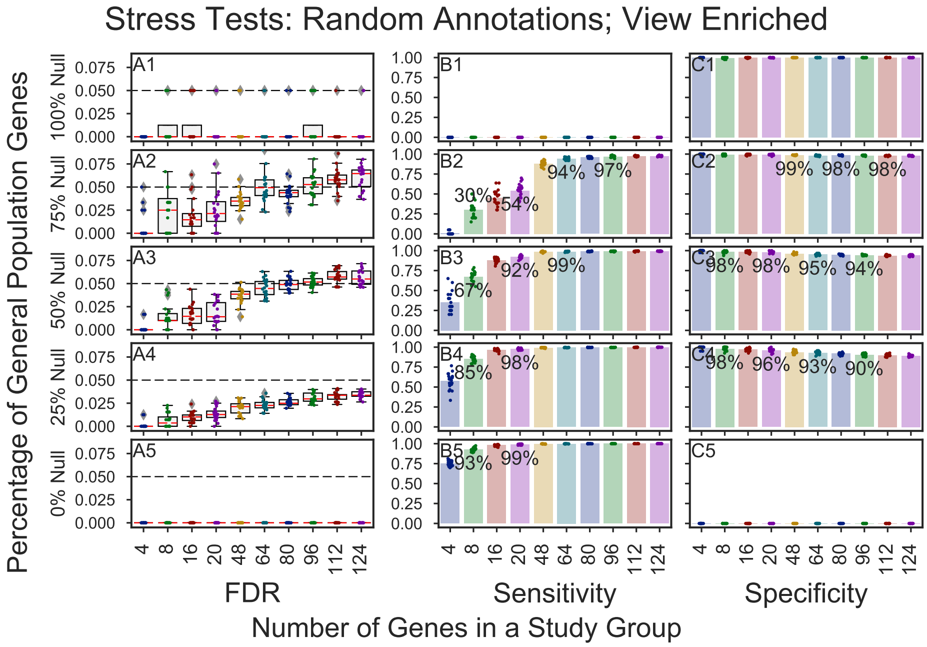

Figure 2. **GOATOOLS GOEAs stress tests with randomly shuffled associations nearly pass if only enriched GO terms are viewed.** The associations are randomly shuffled while still maintaining the distribution number of GO terms per gene. The failing FDRs (above 0.05) are seen in panels A2 and A3 for gene groups having 96, 112, or 124 genes.

Copyright (C) 2009-2018. DV Klopfenstein, H Tang, et al. All rights reserved.

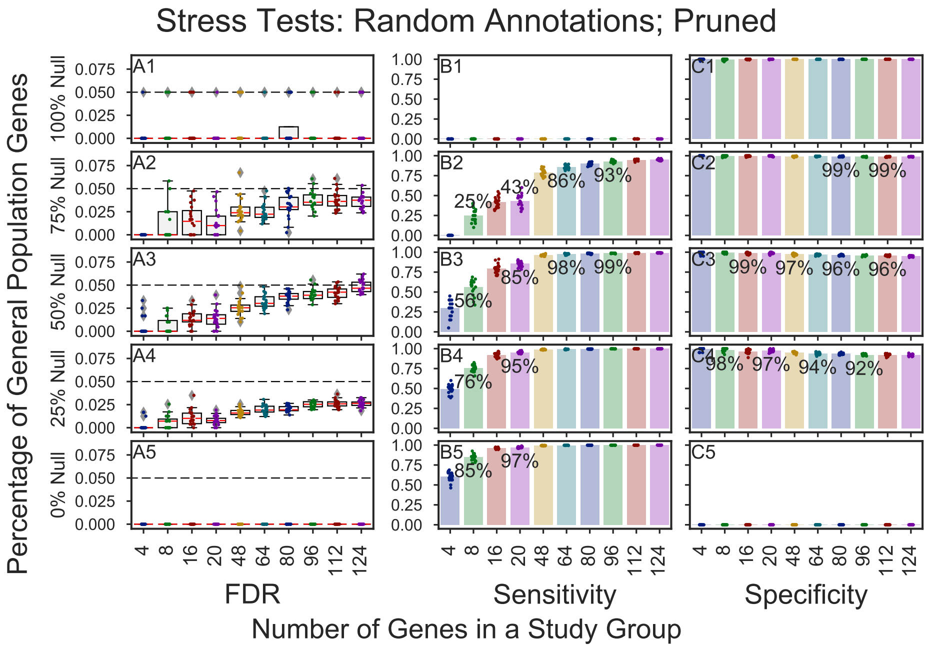

Figure 3. **GOATOOLS GOEAs stress tests with randomly shuffled associations pass for all cases if only 30 out of over 17k+ GO terms associated with more than 1000 genes are removed.** The median number of genes per GO term in the mouse associations is 3 genes/GO. Genes per GO term ranges from 1 gene to 7k genes per GO term. (mean=16 genes/GO, SD=128).

Copyright (C) 2009-2018. DV Klopfenstein, H Tang, et al. All rights reserved.

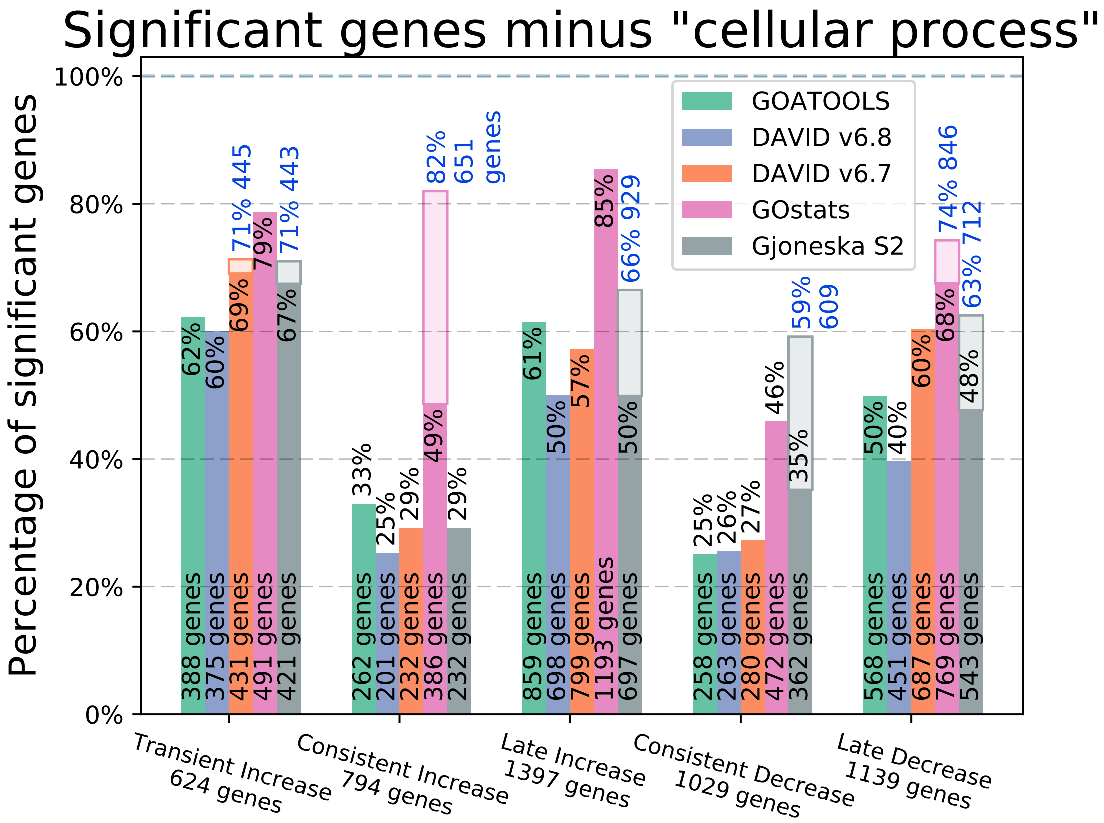

Figure 4. **Removing just one broad GO term can dramatically reduce the percentages of genes associated with statistically significant GO terms.** The GOEA analyses from four different tools plus the Gjoneska DAVID6.7 GOEA results stored in Gjoneska's Supplemental Table 2 found significant GO IDs for five of the six Gjoneska gene clusters using the Gjoneska population background of 13,838 genes. The x-axis shows the five Gjoneska clusters and the total count of genes found to be up or down regulated in the Gjoneska experiments. The solid bars and their black text show the percentage and number of significant genes in the GOEA analyses if *cellular process* is excluded. The faded bars above the solid bars and the associated blue text show the percentage and number of significant genes in the original analyses if *cellular process* is included. The difference in the solid and faded bars shows the affect of removing just one extremely broad GO term, *cellular process*. The color of each bar represents a GOEA tool as specified in the legend. The height of each bar is the percentage of genes in each cluster that are found to be associated with significant GO IDs. It may be desirable to remove GO terms from GEOA analyses that are both broad and are associated with large numbers of genes.

Copyright (C) 2009-2018. DV Klopfenstein, H Tang, et al. All rights reserved.
